# Cumulative live birth rates according to the number of oocytes retrieved following the “freeze-all” strategy

**DOI:** 10.1186/s12958-020-00574-3

**Published:** 2020-02-22

**Authors:** Zhonghua Zhao, Hao Shi, Jing Li, Yile Zhang, Caihong Chen, Yihong Guo

**Affiliations:** 1 Center of Reproductive Medicine, The First Affiliated Hospital of Zhengzhou University, Zhengzhou, 450052 People’s Republic of China; 2 Henan Key Laboratory of Reproduction and Genetics, Zhengzhou, 450052 People’s Republic of China

**Keywords:** Cumulative live birth rate. Ovarian hyperstimulation syndrome. “Freeze-all” strategy. Oocyte number. In vitro fertilization

## Abstract

**Background:**

In recent years, some studies have shown that there is a positive association between the number of oocytes retrieved and the cumulative live birth rate (CLBR) after fresh and frozen cycles of one oocyte retrieval. However, almost no studies have examined the association between the number of oocytes retrieved and the CLBR when using the “freeze-all” strategy. We performed this study to investigate the effects of an extreme oocyte yield during the first “freeze-all” cycle on the cumulative live birth rate among patients younger than 35 years old.

**Methods:**

This was a retrospective cohort study performed in a university-affiliated reproductive medicine centre. Data obtained from 3276 women aged younger than 35 years who underwent their first “freeze-all” cycle (IVF/ICSI) were collected between January 2009 and December 2016. In all, 5025 frozen cycles took place during the follow-up period from January 2009 to December 2018. Patients were divided into five groups according to oocytes retrieved (group 1: 4–10 oocytes; group 2: 11–20 oocytes; group 3: 21–30 oocytes; group 4: 31–40 oocytes; group 5: > 40 oocytes). The primary outcome was the cumulative live birth rate.

**Results:**

Unadjusted results showed that the cumulative live birth rate significantly increased as the number of oocytes retrieved increased and reached up to 93.82% in cases with yields of 21–30 oocytes (*P* < 0.05), after which it did not have a significant increase (*P* > 0.05). After adjusting for confounders, our results showed that the number of oocytes retrieved is an independent positive predictor of cumulative live birth rate when using a “freeze-all” strategy. (*P* < 0.001). In addition, the fertilization rate and the gonadotropin dose also influenced the cumulative live birth rate (*P*<0.05).

**Conclusions:**

Among women younger than 35 years old who underwent the “freeze-all” strategy, the number of oocytes retrieved positively correlated with the cumulative live birth rate. Taking both efficacy and safety into account, ovarian stimulation should be rational, and the upper limit of the oocyte yield should be no more than 30.

## Background

After the advent of controlled ovarian stimulation (COS), multi-follicular stimulation cycles replaced natural mono-follicular cycles in the clinical practice of assisted reproductive technology (ART) [1]. A conventional in vitro fertilization (IVF) procedure starts with COS and ends with the transfer of the best available embryo [1], while all surplus embryos of adequate quality are cryopreserved for later use [2]. However, under some circumstances, a fresh embryo transfer cannot be performed, and the entire cohort of viable embryos is cryopreserved [1]. This is known as the “freeze-all” strategy, in which all embryos are cryopreserved for future frozen-thawed embryo transfer, and this approach is increasingly being favoured [3]. There are many indications for the “freeze-all” strategy: ovarian hyperstimulation syndrome (OHSS), the asynchrony of embryo and endometrial receptivity, preimplantation genetic diagnosis (PGD), preimplantation genetic screening (PGS) [3], fertility preservation and premature progestin elevation [4]. OHSS, an iatrogenic complication of COS accompanied by a higher number of retrieved oocytes, accounts for a substantial proportion of these indications. A strategy comprising the use of a GnRH antagonist for pituitary downregulation, a GnRH agonist for ovulation triggering, and vitrification of all embryos can achieve an OHSS-free status for high responders [3]. In recent years, randomized controlled trials and observational studies have shown that better in vitro fertilization pregnancy outcomes and lower rates of obstetric and perinatal morbidity are achieved following the first frozen-thawed embryo transfer of the “freeze-all” strategy than the fresh embryo transfer [5–7]. Furthermore, some studies have examined the association between the number of oocytes retrieved and the cumulative live birth rate (CLBR) after one fresh and several frozen-thawed embryo transfers following one round of oocyte retrieval, and all of these studies found a positive association [8, 9]. However, almost no study has examined the association between the number of oocytes retrieved and the CLBR when the “freeze-all” strategy is used. Therefore, we carried out the current study to investigate the relationship between the number of oocytes retrieved and the CLBR for the “freeze-all” strategy.

## Methods

### Patients

**Fig. 1:**
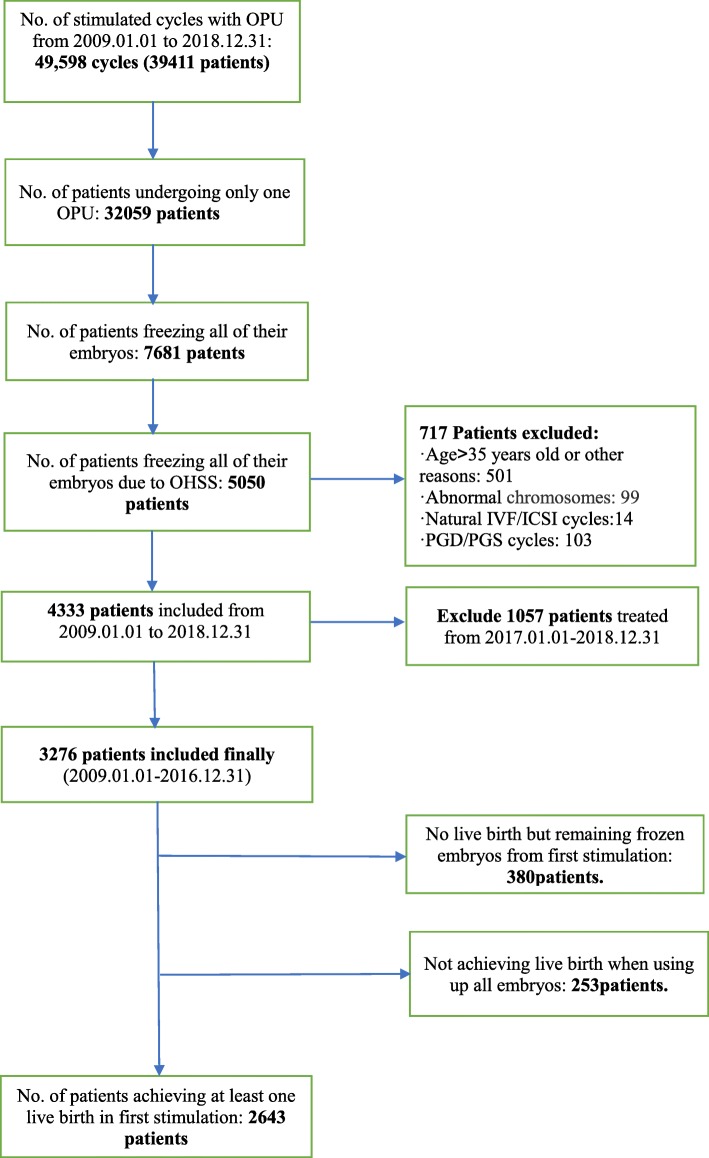
Flowchart of data selection

This retrospective study was based on the Clinical Reproductive Medicine Management System/Electronic Medical Record Cohort Database (CCRM/EMRCD) at the Reproductive Medical Center, First Affiliated Hospital of Zhengzhou University, and the Henan Province Key Laboratory for Reproduction and Genetics. A total of 3276 Chinese women younger than 35 years who underwent a first “freeze-all” strategy were collected between January 2009 and December 2016, and 5025 frozen cycles took place during the follow-up period from January 2009 to December 2018. Of 3276, 380 patients discontinued treatment without a live birth but with embryo(s) left in the first “freeze-all” strategy (we regarded them as dropouts). In our study, only the first “freeze-all” strategy (cycle) due to OHSS tendency or early OHSS, which occurs within 9 days of human chorionic gonadotropin (hCG) administration, was considered. Tubal disease included (1) history of surgery on the fallopian tubes: previous EP, salpingostomy, and tube reconstruction surgery and (2) hysterosalpingography examination: hydrosalpinx and salpingitis. Examples included unilateral or bilateral tubal occlusion, peritubal adhesion, unilateral or bilateral salpingectomy, or tubal ligation. PCOS was diagnosed by the Rotterdam criteria. We excluded donor cycles of oocytes or sperm, natural IVF/ICSI cycles, preimplantation genetic diagnosis (PGD) and preimplantation genetic screening (PGS) cycles. (Fig. 1).

### COS protocols

The protocols were applied based on the day of the patient’s menstrual cycle when the patient came to the hospital. For patients in the follicular phase, triptorelin depot (decapeptyl 3.75 mg; Ipsen Pharma, France) was injected intramuscularly on days 2–3 of the menstrual cycle. Pituitary downregulation was achieved 28–42 days later [FSH (follicle-stimulating hormone) < 4 IU/L, E2 (oestradiol) < 50 pg/ml, LH (luteinizing hormone) < 4 IU/L, endometrium thickness < 5 mm and diameter of the maximal follicle ≤5 mm]. For patients in the luteal phase, triptorelin (Ferring GmbH, 0.1 mg, Germany; Ipsen Pharma Biotech, 0.1 mg, France) was injected intramuscularly during the midluteal phase, and 10 days later, the dose was decreased to 0.05 mg/d until pituitary downregulation [FSH (follicle-stimulating hormone) < 4 IU/L, E2 (oestradiol) < 50 pg/ml, LH (luteinizing hormone) < 4 IU/L, endometrium thickness < 5 mm and diameter of the maximal follicle ≤5 mm] was achieved. Ovarian hyperstimulation was started by FSH (the initial dose was based on patient age, BMI, antral follicle count, AFC, and other factors); the dose of FSH (Gonal-F, Serono, Puregon, Netherlands, u-FSH, Livzon) was adjusted based on follicle development and hormone levels, and human menopausal gonadotropin (HMG, 75 U/ampoule, Ferring GmbH, Germany), was added if needed. When the maximal follicle was more than 20 mm and more than 2/3 follicles > 16 mm, human chorionic gonadotropin (2000 IU) (HCG, Lizhu. Ltd., Guangdong, China) and recombinant HCG (250 μg) (Merck Serono, Italy) were administered to trigger oocyte maturation. Approximately 36–37 h after hCG was administered, oocyte retrieval was performed with transvaginal ultrasound guidance. IVF or ICSI was performed based on sperm parameters. Patients at risk of ovarian hyperstimulation syndrome (OHSS) were cancelled for embryo transfer and underwent whole-embryo cryopreservation. Luteal phase was supported with 60 mg progesterone (20 mg/ampoule, Xianju Ltd., Zhejiang, China) from the day of ovum pick-up (OPU).

### Endometrial preparation schemes

The endometrial preparation schemes for frozen embryo transfer (FET) in the current study were natural cycles and artificial (Oestrogen–Progesterone) cycles. For natural cycles, an ultrasound was performed on day 8–9 of the menstrual cycle to evaluate the endometrial thickness and the diameter of the dominant follicle. When the dominant follicle was 16–20 mm, serum progesterone and LH levels were monitored to confirm the ovulation time. Thawing and transferring were performed 3 days after ovulation. Luteal phase support was sustained with intramuscular progesterone 40 mg (20 mg/ampoule, Xianju Ltd., Zhejiang, China) administered on the day of ovulation and oral dydrogesterone 20 mg (Solvay Pharmaceuticals B.V., Veenendaal, The Netherlands) administered on the transfer day. For Oestrogen-Progesterone cycles, 2–4 mg/day of oral oestradiol ([Progynova]; Bayer, Germany) was given on day 3 of the menstrual cycle, and the dose was adjusted based on endometrial thickness. After 12–14 days, if no leading follicle was present, intramuscular progesterone 60 mg (20 mg/ampoule, Xianju Ltd., Zhejiang, China) and oral dydrogesterone 10 mg or 20 mg (Solvay Pharmaceuticals B.V., Veenendaal, The Netherlands) were given. Embryo transfer was performed 3 days later.

### Outcomes

The primary outcome was the cumulative live birth per started cycle, which was defined as at least one liveborn baby at 24 weeks of gestation resulting from an ART-initiated cycle. Clinical pregnancy was defined as at least one gestational sac with or without foetal heart activity by ultrasound at 4–5 weeks after embryo transfer. Live birth was defined as one or more live babies born after 24 weeks of gestation.

### Statistical analysis

Statistical analyses were performed with SPSS (Statistical Package for Social Science, SPSS Inc., Chicago, IL, USA) version 19.0. Patients were classified as live birth or no live birth and were then stratified according to the number of oocytes retrieved: 4–10, 11–20, 21–30, 31–40, or > 40 oocytes. We performed Shapiro-Wilk tests for continuous variables, and none of them fulfilled the criteria for normal distribution. Continuous variables are expressed as the medians (interquartile ranges, IQR) compared via the Mann-Whitney test (two groups) or Kruskal-Wallis test (more than two groups). Categorical variables are presented as percentages compared via the chi-square test. The differences between groups were compared using Bonferroni correction, and *P* < 0.05/10 was set as indicating a significant difference. Multivariate logistic regression analyses were performed to explore the association between the number of retrieved oocytes and the CLBR. The results are presented as the adjusted odds ratios (aORs) with the 95% confidence intervals (CIs). Differences were considered significant if *P* < 0.05.

## Results

**Fig. 2:**
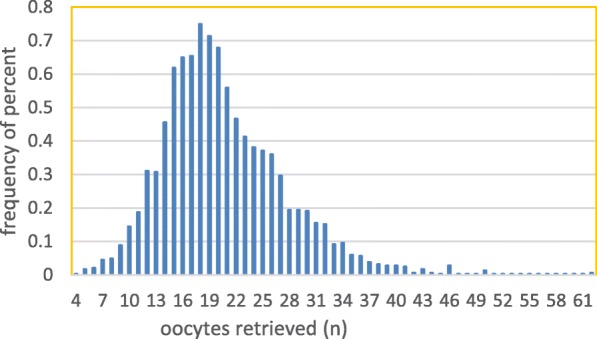
Distribution of oocyte yield

In the current study, the median number of oocytes retrieved was 20 (interquartile range 16–24), and the distribution of aspirated oocytes is shown in Fig. 2. The median age of the women was 28 (interquartile range 25–30) years. The overall CLBR was 91.26%, and the cumulative clinical pregnancy rate (CCPR) was 93.68%.

### Baseline characteristics and treatment outcomes of women with or without live birth

Table 1 shows that the two groups had the same baseline characteristics in terms of age, BMI, basal FSH, basal LH, diagnosis of infertility, insemination method, stimulation protocols, and peak E2 on HCG day (*P* > 0.05). The women who achieved a live birth had a significantly higher basal AFC and significantly higher fertilization rates and numbers of oocytes retrieved than were found in those without a live birth (*P* < 0.05). However, we found that women who achieved a live birth required a lower gonadotropin dose than was used in women without live a birth (*P* < 0.05).

**Table 1:** Baseline characteristics and treatment outcomes of women with or without live birth

Variables	Live birth (*N* = 2643)	No live birth (*N* = 253)	*P*
Age (years)	28.0(25–30)	27.5(25–31)	NS
Body mass index (kg/m^2^)	21.9(20.0–24.2)	22.0(19.9–24.4)	NS
Basal follicle-stimulating hormone (IU/L)	5.97(5.09–7.0)	6.11(5.01–7.15)	NS
Basal luteinizing hormone (IU/L)	5.69(4.25–8.16)	5.50(4.21–7.60)	NS
Basal antral follicular count (n)	18(13–24)	17(11–24)	**0.031**
Cause of infertility			NS
Male factor	26.0% (686/2643)	25.3% (64/253)	
Tubal disease	39.0% (1032/2643)	41.5% (105/253)	
PCOS	13.7% (363/2643)	9.9% (25/253)	
Endometriosis	1.3% (33/2643)	0.8% (2/2530)	
Unexplained	20.0% (529/2643)	22.5% (57/253)	
Insemination method			NS
IVF	69.7% (1838/2643)	67.6% (171/253)	
ICSI	30.3% (798/2643)	32.4% (82/253)	
Stimulation protocols			NS
Long-acting GnRH-a long protocol	35.4% (936/2643)	33.2% (84/253)	
Short-acting GnRH-a long protocol	63.5% (1647/2643)	65.2% (165/253)	
Unrecorded	1.1% (33/2643)	1.6% (4/253)	
Gonadotropin dosage (IU)	1537(1237–1987)	1650(1331–2250)	**0.002**
Fertilization rate(%)	0.69(0.57–0.80)	0.61(0.46–0.73)	**0.000**
Peak E_2_ on HCG day (IU/L)	7276(4991–9484)	7084(4448–9103)0	NS
No. of oocytes retrieved(n)	20(16–25)	18(14–21)	**0.000**

Note: Data are presented as medians (interquartile ranges, IQRs) and percentiles (numbers). *P* < 0.05 was set as indicating a significant difference

*PCOS* Polycystic ovary syndrome, *IVF* In vitro fertilization, *ICSI* Intracytoplasmic sperm injection, *E*_*2*_ Oestradiol, *NS* nonsignificant, defined as *P* ≥ 0.05

The bold values indicated that the difference was significant

### IVF/ICSI outcome of each oocyte-stratified group in the “freeze-all” strategy analysis

Table 2 shows that there was no significant difference in age among the five groups (*P* = 0.507). However, groups 1 and 2 had significantly higher basal FSH levels and fertilization rates than were found in groups 3, 4, and 5. Groups 3, 4, and 5 had significantly higher peak E2 on HCG day, basal AFC and BMI than were found in groups 1 and 2. Group 2 had significantly lower basal LH levels and gonadotropin dosages than were found in group 4. The results of the Bonferroni pairwise comparison showed that the CLBR of group 1 was significantly lower than those of groups 2, 3, 4, and 5 (*P*<0.001) and especially group 2 (72.48% vs. 90.28%). Additionally, the CLBR was significantly lower in group 2 than in group 3 (90.28% vs. 93.82%, *P*<0.005). However, no significant difference was found in the CLBR among group 3, 4, and 5 (93.82% vs. 95.26% vs. 97.22%, *P* > 0.005). We obtained the same results when we analysed the CCPR.

**Table 2:** IVF/ICSI outcomes for each oocyte-stratified group

Variables	Group 1 (*N* = 109)	Group 2 (*N* = 1553)	Group 3 (*N* = 987)	Group 4 (*N* = 211)	Group5 (*N* = 36)
Age (years)	28(25–31)	28(26–30)	28(25–30)	28(25–30)	27(25–30)
BMI (kg/m^2^)	20.8(19.3–23.0) ^bcd^	21.5(19.6–23.9) ^αβ^	22.0(20.3–24.6)	22.6(20.8–25.0)	23.4(20.8–26.6)
Basal FSH (IU/L)	6.4(5.24–7.45) ^bcd^	6.1(5.22–7.14) ^αβγ^	5.7(4.95–6.76)	5.6(4.82–6.48)	5.5(4.63–6.36)
Basal LH (IU/L)	5.57(3.98–7.22)	5.53(4.18–7.78) ^β^	5.72(4.29–8.49)	6.43(4.55–9.09)	6.93(4.70–8.67)
Peak E2 on HCG day (IU/L)	6358(3425–8676) ^bcd^	6966(4754–9122) ^αβγ^	7778(5314–9732)	8038(5477–10,929)	8863(5696–11,939)
Basal AFC (n)	14(10–20)^bcd^	17(12–22) ^βγ^	20(14–24)	22(15–24)	24(22.5–24)
Gn dosage (IU)	1575(1231–1925)	1537(1237–1950) ^β^	1575(1275–2062)	1637(1312–2387)	1631(1406–2493)
Fertilization rate (%)	0.70(0.60–0.88) ^bcd^	0.71(0.57–0.81) ^αβ^	0.67(0.55–0.76)	0.67(0.56–0.75)	0.62(0.53–0.74)
CCPR [%(n/N)]	81.65(89/109) ^abcd^	92.85(1442/1553) ^α^	95.74(945/987)	95.73(202/211)	97.22(35/36)
CLBR [%(n/N)]	72.48(79/109) ^abcd^	90.28(1402/1553) ^α^	93.82(926/987)	95.26(201/211)	97.22(35/36)

Note: Data are presented as medians (interquartile ranges, IQRs)

*BMI* Body mass index, *FSH* Follicle-stimulating hormone, *LH* Luteinizing hormone, *AFC* Antral follicular count, *Gn* gonadotropin*, CCPR* Cumulative clinical pregnancy rate, *CLBR* Cumulative live birth rate, *NS* nonsignificant, defined as *P* ≥ 0.05

The differences between groups (Bonferroni correction, *P* < 0.05/10) are indicated by superscripts:

^a^*P*, ^b^*P*, ^c^*P* and ^d^*P* indicate group 1 vs. groups 2, 3, 4, and 5, respectively

^α^*P*, ^β^*P* and ^γ^*P* indicate group 2 vs. groups 3, 4and 5, respectively

^†^*P* and ^‡^*P* indicate group3 vs. groups 4 and 5, respectively

^*^P indicates group 4 vs. group 5

### Multivariate logistic regression analysis of the CLBR for the “freeze-all” strategy

Table 3 shows the multilevel logistic regression results for the number of oocytes retrieved and the CLBR. Group 1 (4–10 oocytes) was used as a reference. After adjusting for female age, BMI, basal AFC, FSH, LH levels, gonadotropin dose, peak E2 on HCG day, stimulation protocols, insemination method, and fertilization rate, the number of oocytes retrieved remained an independent positive predictor (*P*<0.001) of CLBR. The aORs and 95% CIs for the CLBR increased from 3.987 (2.411–6.564) in group 2 to 6.851 (3.924–11.960) in group 3, 9.579 (4.122–22.259) in group 4 and 18.378 (2.312–146.101) in group 5. The fertilization rate positively affected the CLBR, while the gonadotropin dose negatively affected the CLBR (*P*<0.05).

**Table 3:** Multivariate logistic regression analysis of the CLBR for the “freeze-all” strategy

Predictors	aOR (95% CI)	*P*
Age	1.007(0.963–1.054)	0.749
Body mass index	1.003(0.955–1.052)	0.961
Basal antral follicular count	1.004(0.978–1.031)	0.764
Basal follicle-stimulating hormone	1.029(0.951–1.113)	0.474
Basal luteinizing hormone	1.016(0.983–1.050)	0.347
Log_10_ (Gn) dosage	0.195(0.059–0.640)	**0.007**
E_2_ on HCG day	0.958(0.478–1.920)	0.903
Stimulation protocols		0.108
Long-acting GnRH-a long protocol	1	
Short-acting GnRH-a long protocol	0.686(0.473–0.994)	0.047
Unrecorded	1.306(0.366–4.659)	0.680
Insemination method		
IVF	1	
ICSI	0.808(0.596–1.097)	0.172
Oocyte yield		**0.000**
4–10	1	
11–20	3.987(2.411–6.564)	**0.000**
21–30	6.851(3.924–11.960)	**0.000**
31–40	9.579(4.122–22.259)	**0.000**
≥ 40	18.378(2.312–146.101)	**0.006**
Fertilization rate	16.877(8.100–35.166)	**0.000**

*Note*: Data are presented as aOR (95% CI); *P* < 0.05 was set as indicating a significant difference

*E*_*2*_ Oestradiol, *IVF* In vitro fertilization, *ICSI* Intracytoplasmic sperm injection; *Log*_*10*_*(Gn) dose* a transformation of gonadotropin dose, *CI* Confidence interval

The bold values indicate that the differences are significant

Table 4 shows that 380 patients discontinued fertility treatment at the end of follow-up. The total dropout rate was 11.5%. We analysed these patients according to the number of attempts at embryo transfer. The results showed that among the first three groups, as the number of oocytes yielded increased, the number(s) of attempted embryo transfers increased, the ratio of the number of embryos used to the number of oocytes yielded increased, and the ratio of the number of oocytes left to the number of oocytes yielded decreased.

**Table 4:** Patients who discontinued treatment with no live birth but with remaining frozen embryo(s)

Variables	Number of attempt(s)				
	1 (*N* = 117)	2 (*N* = 151)	3 (*N* = 77)	4 (*N* = 26)	5 (*N* = 9)
Age (years)	28(26–30)	28(26–31)	29(27–31)	29(26–31)	27(24–28)
Body mass index (kg/m^2^)	22.5(20.0–25.4)	22.3(20.2–25.7)	22.6(20.3–25.4)	24.9(21.3–26.8)	24.0(20.1–26.6)
No. of oocytes yielded (n)	6(5–9) ^ab^	8(6–11) ^α^	10(8–13)	13(10–18)	15(11.5–16)
No. of embryos used / No. of oocytes yielded (%)	0.33(0.25–0.50) ^ab^	0.57(0.40–0.71) ^α^	0.73(0.55–0.83)	0.73(0.64–0.88)	0.92(0.80–0.93)
No. of embryos left / No. of oocytes yielded (%)	0.67(0.50–0.75) ^ab^	0.43(0.29–0.60) ^α^	0.27(0.17–0.45)	0.27(0.13–0.26)	0.08(0.07–0.20)

*Note*: Data are presented as medians (interquartile ranges, IQRs)

The differences between groups (Bonferroni correction, *P* < 0.05/10) are indicated by the following superscripts:

^a^*P*, ^b^*P*, ^c^*P* and ^d^*P* indicate group 1 vs. groups 2, 3, 4, and 5, respectively

^α^*P*, ^β^*P* and ^γ^*P* indicate group 2 vs. groups 3, 4 and 5, respectively

^†^*P* and ^‡^*P* indicate group 3 vs. groups 4 and 5, respectively

^*^P indicates group 4 vs. group 5

## Discussion

The present study shows that among patients younger than 35 years old, the number of oocytes retrieved was an independent positive predictor of the CLBR in patients using the “freeze-all” strategy. The chance of having a live birth was 91.26% after a completed “freeze-all” cycle.

The impact of a higher number of oocytes retrieved after ovarian stimulation has been investigated in many prior studies. Advocates of mild stimulation suggest that obtaining fewer oocytes could achieve a lower embryo aneuploidy rate and a better implantation rate [10, 11]. In contrast, other studies have reported that the numbers of usable blastocysts and euploid embryos increased with the number of oocytes retrieved [12]. These findings may explain the positive association between the number of oocytes retrieved and the CLBR. Consistent with these findings, in our study, the fertilization rate was lower, but the CLBR and CCPR were higher in groups 3, 4, and 5 than in groups 1 and 2. Our findings also showed that groups 3, 4, and 5 had higher basal FSH, basal AFC and peak E2 levels on HCG day than were found in groups 1 and 2. Similar results were found by Fatemi et al. [9]. In our study, although the included patients either showed a tendency to have OHSS or had OHSS, we eliminated the influence of this factor by administering hydroxyethyl starch and albumin as a plasma expander for several days and cancelling the fresh embryo transfer by freezing all usable embryos. These measures can bypass the detrimental effects of supraphysiological oestradiol levels and improve endometrial receptivity in a deferred frozen-thawed embryo transfer.

To date, studies investigating the association between the number of oocytes retrieved and the CLBR for the “freeze-all” strategy were scarce. However, we found ten studies to date that evaluated the association between the number of oocytes retrieved and CLBR following a conventional in vitro fertilization strategy, which consists of a single fresh and all frozen-thawed embryo transfers after one aspiration, and all found a positive correlation between the number of oocytes retrieved and the CLBR [8, 9, 12–15]. Apart from showing a positive correlation, some of these ten studies determined what oocyte number was ideal for the optimal CLBR. Magnusson et al. suggested that retrieving 18 oocytes would be optimal for cumulative live birth in patients of all ages [16]. Without mentioning age, Chen et al. from China indicated that when the oocyte number was more than 10 for PCOS patients, the CLBR did not increase [17]. A paper published in *Human Reproduction* showed that the ideal number of retrieved oocytes was approximately 25 for the optimal CLBR among women aged 18–35 years, while retrieving more than 30 oocytes was optimal in women aged 36–44 years, and retrieving 9 oocytes was ideal for women older than 45 years [18]. Although there was a great deal of heterogeneity in parameters such as sample size, patient age, study design, stimulation protocols et al. among these ten studies, they convinced us that there is a strong positive correlation between the number of oocytes retrieved and the CLBR in conventional in vitro fertilization strategies. However, none of these ten studies analysed the CLBR for “freeze-all” strategy. On the basis of the ten previous studies, our study further enriches the field and confirms that the same pattern exists for the “freeze-all” strategy: unadjusted results showed that the CLBR significantly increased as the number of oocytes retrieved increased and reached as high as 93.8% when 21–30 oocytes were aspirated and then did not have a significant increase. Multivariate logistic regression revealed that the number of oocytes retrieved remained an independent predictive factor for CLBR. Our findings are supported by a few studies investigating the “freeze-all” strategy. A prospective cohort observational study showed that in the “freeze-all” cycle, a higher number of frozen blastocysts was associated with a higher CLBR in women younger than 40 years old with a normal/high response and that the highest CLBR, 88%, occurred when more than 7 blastocysts were frozen [19]. However, that study was limited by its small sample size of 254 patients. Another retrospective cohort study showed that the CLBR improved as the number of oocytes yielded increased up to 25 oocytes when using the “freeze-all” strategy [20]. However, the author did not clarity the reason why all the embryos were frozen in his study. Freezing all embryos due to fertility preservation and due to OHSS are two completely different situations. Our study eliminated this heterogeneity by including the OHSS-only patients and showed that the upper limit for the number of oocytes retrieved should be no higher than 30 when using the “freeze-all” strategy. Our study also shows that the fertilization rate positively affected while the gonadotropin dosage negatively affected the CLBR. These findings are consistent with previous studies [13, 21].

The dropout rate in our study was 11.5%. Among the patients who discontinued, we found that as the number of oocytes increased, the attempted number of embryo transfers increased, the ratio of the number of embryos used to the number of oocytes yielded increased, and the ratio of the number of oocytes left to the number of oocytes yielded decreased. These findings were consistent with a previous study in which patients in whom fewer oocytes were retrieved were more likely to discontinue further treatment due to the couple’s perception of their poor treatment prognosis and the psychological burden [22].

Given that the patients in our study were younger than 35 years old, we cannot provide any guidance for patients of advanced age. Although we tried our best to reduce selection and confounding biases, there are still inherent biases related to the retrospective design of this study; these include changes in clinical practice that occurred during the long study period. Finally, the number of patients in whom more than 40 oocytes were retrieved was small (*N* = 36). However, this still represents the real number of oocytes retrieved, as a yield of more than 40 oocytes is rare in the real world. Despite the above limitations, we believe that our study has significant implications for clinicians and patients who must make informed decisions regarding the “freeze-all” strategy. However, we do not recommend that an extreme oocyte yield should be a goal given the risks of ovarian torsion, venous thrombo-embolism and arterial thrombosis, particularly because achieving an oocyte retrieval number higher than 30 is pointless when using the “freeze-all” strategy. This may be because younger women should have a sufficient number of euploid embryos to provide multiple possibilities for achieving a live birth, and the surplus embryos would therefore be wasted in these individuals [15].

## Conclusion

In conclusion, when using the “freeze-all” strategy, the number of oocytes retrieved is a positive predictor of the CLBR among women less than 35 years old. Given that the CLBR significantly increased with the number of oocytes retrieved, reaching as high as 93.82% when 21–30 oocytes were aspirated, the ovarian stimulation should be rational, and the upper limit of the oocyte yield should be no more than 30 to avoid severe iatrogenic complications.

## Data Availability

The datasets used in the current study are available from the corresponding author on reasonable request.

## Abbreviations

AFC: Antral follicular count
ART: Assisted reproductive technology
BMI: Body mass index
CCPR: Cumulative clinical pregnancy rate
CI: Confidence interval
CLBR: Cumulative live birth rate
COS: Controlled ovarian stimulation
FSH: Follicle-stimulating hormone
ICSI: Intracytoplasmic sperm injection
IVF: In vitro fertilization
LH: Luteinizing hormone
OHSS: Ovarian hyperstimulation syndrome
OR: Odds ratio
PCOS: Polycystic ovary syndrome
PGD: Preimplantation genetic diagnosis
PGS: Preimplantation genetic screening
